# Boron-Doped Nanocrystalline Diamond Electrodes for Neural Interfaces: *In vivo* Biocompatibility Evaluation

**DOI:** 10.3389/fnins.2016.00087

**Published:** 2016-03-08

**Authors:** María Alcaide, Andrew Taylor, Morten Fjorback, Vladimir Zachar, Cristian P. Pennisi

**Affiliations:** ^1^Laboratory for Stem Cell Research, Department of Health Science and Technology, Aalborg UniversityAalborg, Denmark; ^2^Institute of Physics, Academy of Sciences of the Czech Republic v.v.iPrague, Czech Republic; ^3^Nano6 s.r.o.Kladno, Czech Republic; ^4^Neurodan A/SAalborg, Denmark

**Keywords:** nanocrystalline diamond, neuroprosthetic interfaces, neural electrodes, boron-doped diamond, titanium nitride, foreign body reaction, biocompatible materials, *in vivo* models

## Abstract

Boron-doped nanocrystalline diamond (BDD) electrodes have recently attracted attention as materials for neural electrodes due to their superior physical and electrochemical properties, however their biocompatibility remains largely unexplored. In this work, we aim to investigate the *in vivo* biocompatibility of BDD electrodes in relation to conventional titanium nitride (TiN) electrodes using a rat subcutaneous implantation model. High quality BDD films were synthesized on electrodes intended for use as an implantable neurostimulation device. After implantation for 2 and 4 weeks, tissue sections adjacent to the electrodes were obtained for histological analysis. Both types of implants were contained in a thin fibrous encapsulation layer, the thickness of which decreased with time. Although the level of neovascularization around the implants was similar, BDD electrodes elicited significantly thinner fibrous capsules and a milder inflammatory reaction at both time points. These results suggest that BDD films may constitute an appropriate material to support stable performance of implantable neural electrodes over time.

## Introduction

In recent years boron-doped nanocrystalline diamond (BDD) has become an established electrode material for electrochemical applications due to its many outstanding properties, which include high corrosion resistance, a wide potential window of water stability, and low background currents (Park et al., 2005; Luong et al., 2009; Roeser et al., 2013). In the context of *in vivo* biomedical applications, BDD electrodes have been primarily applied in the development of biochemical sensors exhibiting high precision and stability for the detection of neurotransmitters and various other biomolecules (Suzuki et al., 2007; Fierro et al., 2012, 2013). The capability of BDD electrodes to measure bioelectrical activity and provide neural stimulation have been first explored by Halpern et al. using the marine mollusk *Aplysia californica* as a neural circuit model (Halpern et al., 2006, 2010). Further studies have shown the feasibility of using conductive diamond for the acute measurement of bioelectric potentials from mammalian neural cells, but the recording performance of the electrodes in these studies was limited due to low double layer capacitances and high impedances (Ariano et al., 2009; Chan et al., 2009). In recent years, diverse nanomaterials have emerged as means to increase the electrochemically active surface area of neural electrodes, allowing the fabrication of microelectrodes with superior electrochemical performance as compared to the unmodified counterparts (Boehler et al., 2015; Kim et al., 2015). This new generation of microelectrodes, in perspective, may allow the development of novel neural prostheses possessing high sensitivity and spatial resolution. Similarly, studies have indicated that the limitations of BDD may be overcome by providing a nanostructured surface onto which the diamond films are grown (Hébert et al., 2014; He et al., 2015; Meijs et al., 2015b). The specific increase in surface area brought by the three-dimensional nanostructures has demonstrated a significant improvement in neural recording and stimulation capabilities of BDD microelectrodes in neural tissue preparations (Piret et al., 2015). These recent advances have contributed to the increased interest in BDD electrodes for electrical interfacing with neural cells, such as implantable neural prostheses and brain-computer interfaces.

While BDD electrodes appear to be well-suited for acute neural interfacing applications, it remains unknown if chronically implanted BDD electrodes are able to efficiently record and/or activate neurons over time. An essential requirement to ensure a stable long-term performance in implantable neural devices is an interface that minimizes the healing response to implantation known as the foreign body reaction (Merrill, 2014). The presence of encapsulation tissue seriously compromises the quality of signals recorded from electrodes chronically implanted in the brain and peripheral nerves (Grill and Mortimer, 1994; Marin and Fernández, 2010). In neural stimulation applications, the encapsulation tissue increases the demand of charge needed for cell activation, which might cause irreversible damage to the electrode and tissue (Merrill et al., 2005). Although, the biocompatibility of undoped diamond films has been addressed by several studies (Tang et al., 1995; Amaral et al., 2008; Smisdom et al., 2009; Kloss et al., 2011), reports concerning the biological effects of highly doped BDD materials are still scarce. The early investigations have been focused on the assessment of bone-derived cell cultures on BDD films, showing that BDD surfaces do not exhibit cytotoxicity, support adhesion, proliferation, and osteogenic differentiation of the cells (Kopecek et al., 2008; Kromka et al., 2010; Grausova et al., 2011). Recent studies have addressed the biocompatibility of BDD using neural cell cultures, showing that BDD surfaces are suitable for adhesion and proliferation of neuroblastoma cell lines (Vaitkuviene et al., 2015) and human neural stem cells (Taylor A. C. et al., 2015). In addition, the *in vivo* biocompatibility of BDD has been recently assessed using BDD coated disks subcutaneously implanted in guinea pigs (Garrett et al., 2016). BDD implants elicited the formation of thin fibrous capsules, evidencing a soft tissue response that was milder than that obtained using silicone polymer disks.

Our aim is to investigate the performance of BDD neural electrodes, which belong to a system designed for the treatment of urinary incontinence through a minimally invasive implantation procedure (Martens et al., 2010). In preliminary experiments, we have assessed *in vitro* the electrochemical properties of these BDD electrodes (Meijs et al., 2013). As compared to electrodes coated with smooth titanium nitride (TiN), BDD electrodes displayed similar charge injection capacity, a larger charge storage capacity, and a wider potential window (Meijs et al., 2013). The aim of this study is to describe the surface properties of BDD neural electrodes and to investigate the *in vivo* biocompatibility of these electrodes using a rat subcutaneous implantation model. The biological performance of BDD *in vivo* is assessed in relation to conventional TiN electrodes.

## Materials and methods

### Implant fabrication

**Figure 1:**
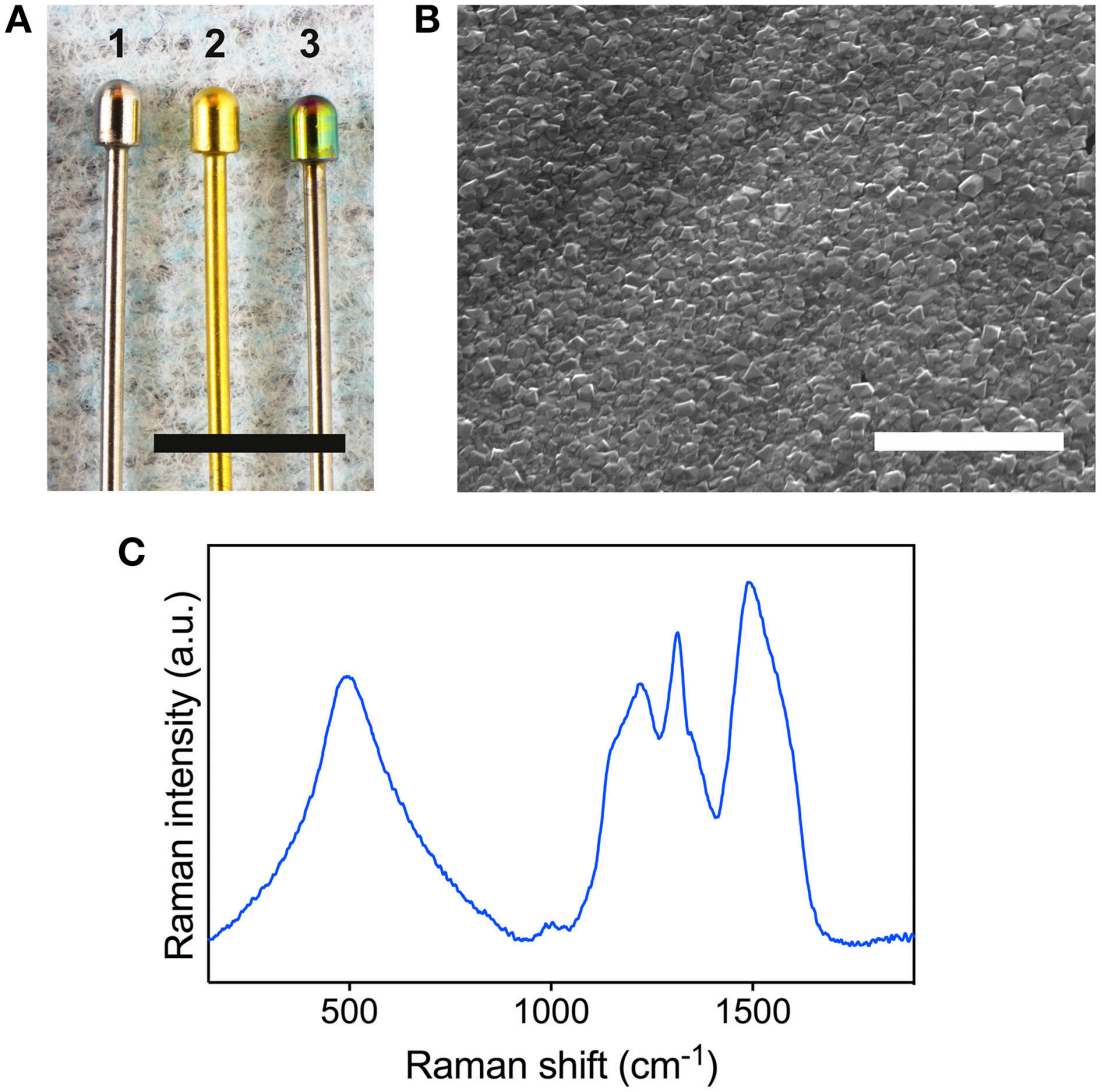
**Surface characterization of the BDD electrodes. (A)** The three types of implants used in this study are displayed for comparison: bare Ti (1), TiN coated (2), and BDD coated implant (3). Scale bar denotes 5 mm. **(B)** Scanning electron microscopy image of the surface of a BDD implant. Scale bar denotes 2 μm. **(C)** 488 nm Raman spectrum obtained from a BDD implant.

The test implants consisted of metallic contacts of a monopolar extraneural electrode, which is intended for genital nerve stimulation for the treatment of urinary incontinence (Meijs et al., 2014). The metallic contact is made of Ti6Al4V alloy grade 5 (ELOS Medtech Pinol A/S, Denmark) and comprise of a stem and a semi-spherical head with a surface area of 6 mm^2^. To fabricate the BDD implants, the electrode heads were seeded with a nanodiamond dispersion with an average mean crystal size of 4–6 nm (NanoAmando®B, NanoCarbon Research Institute Ltd., Japan). BDD films were grown using a microwave plasma enhanced chemical vapor deposition (CVD) apparatus with linear antenna delivery system operating at low pressures with a CH_4_ /H_2_ gas mixture (2.5% CH_4_ + 97.5% H_2_) with trimethylboron as a boron dopant (B/C = 15000 ppm; Taylor et al., 2014). The TiN coatings were applied to the implants using magnetron sputtering, following a method that has been previously described (Meijs et al., 2015a). Uncoated Ti6Al4V implants were used as controls (designated as Ti implants). Figure 1A displays the final aspect of the three types of implants used in this study.

### Implant surface characterization

Scanning electron microscopy (SEM) was performed to investigate the morphology of the BDD films using a Tescan FERA 3 tool (Tescan, Brno, Czech Republic). Raman spectroscopy was carried out at room temperature using a Renishaw inVia Raman microscope at a wavelength of 488 nm and a laser power of 6 mW at the sample.

### Animal model and implantation procedure

All *in vivo* experimental procedures were carried out according to the national laws and guidelines concerning animal experimentation. The study protocol was approved by the Danish Animal Experiments Inspectorate.

Twelve adult Wistar rats (250–300 g) were used for this study. The animals were kept in separate cages, with food and water *ad libitum*. Animals were anesthetized by a subcutaneous injection of 0.2 ml/100 g body weight of a mixture of Hypnorm (VetaPharma) and Dormicum (Accord Healthcare). The dorsum of the rats was shaved and cleaned. Four sterilized electrode pins were implanted on each rat through incisions that were made in the skin along the midline using an 18 G needle. A total of 16 electrodes were implanted for each material type, eight for each time point. The wounds were closed using a non-absorbable nylon suture by a single surgical knot. The animals were monitored on a daily basis for indications of infection, abnormal wound healing or unusual behavior.

### Histological examination

After either 2 or 4 weeks of implantation, animals were euthanized by CO_2_ inhalation. For the histological assessment, the tissue surrounding the implants were cut and immersed in 10% buffered formalin. After 1 week of fixation, the implants were carefully removed and the tissue samples were sectioned, processed and paraffin embedded. The tissue samples were sectioned at the locations that contained the electrode heads, perpendicularly to the main axis of the implants, as depicted in the Supplementary Figure 1. Histological sections (4–5 μm thick) were stained with hematoxylin and eosin (HE) and Masson's trichrome (MT). Stained sections were observed under an inverted microscope using ×10 and ×20 objectives (Axio Observer.Z1, Carl Zeiss) and images were acquired using a digital color camera (Axio Cam MRc, Carl Zeiss). Using the MT images taken at ×20, the thickness of the collagen capsule around each implant was measured at multiple random locations on the implant interface using ImageJ (NIH, Bethesda, MD). In addition, for a semi-quantitative assessment of the local effects of the implants, 12 fields for each implant type per time point were acquired from the HE stained sections at higher magnification (×40). The inflammatory response was determined by assessing the presence of polymorphonuclear cells, lymphocytes, plasma cells, macrophages, giant cells, and necrosis. Neovascularization scores were obtained quantifying the number of blood vessels adjacent to the interface. Scores were given for each image based on the criteria presented as supplementary information (Supplementary Tables I, II). The researcher scoring the images was blinded in regards to the identity of the images.

### Statistical analysis

Capsule thickness values were compared using Kruskal Wallis' non-parametric test with Dunn's multiple comparison in GraphPad Prism 6 (GraphPad Software, La Jolla, CA). Inflammation and vascularization scores were analyzed by ordinal logistic regression using SPSS Statistics v21 (IBM), in which the covariates were the material types and the time. Statistical significance was assigned to differences with *P* < 0.01, unless otherwise specified.

## Results

### Quality assessment of the diamond films

The quality of the BDD surfaces was assessed using SEM and Raman spectroscopy. A typical SEM micrograph of the BDD film grown on the electrode head is displayed in Figure 1B. The surface of the deposited BDD films consisted on a dense array of sharp-edged diamond crystallites with random crystallographic orientation. Raman spectroscopy revealed good homogeneity across all samples. Figure 1C shows a representative spectrum obtained from a BDD film grown on the electrode head. The diamond related sp^3^ peak is observed down shifted from its usual 1332 cm^−1^ position due to boron incorporation. Also present are broad features at 1150 and 1490 cm^−1^ that are generally accepted as originating from transpolyacetylene lying in grain boundaries (Ferrari and Robertson, 2001). In addition, bands at 500 and 1230 cm^−1^ related to boron incorporation are present. Semi-quantitative analysis of the Raman spectra revealed an average sp^3^/sp^2^ (diamond/graphite) ratio of 94% and an average boron incorporation of 4.4E + 21 cm^−3^ in the BDD layers.

### General outcome of the *in vivo* study

During the course of the *in vivo* experiments, no signs of infection, abnormal wound healing, or unusual behavior was observed in any of the experimentation animals. At the time of implant retrieval, macroscopic analysis of the tissue around the implants revealed good quality healing and no evidence of wound dehiscence. An image of a BDD electrode after 2 weeks of implantation is shown in the Supplementary Figure 1.

### Histological analysis

**Figure 2:**
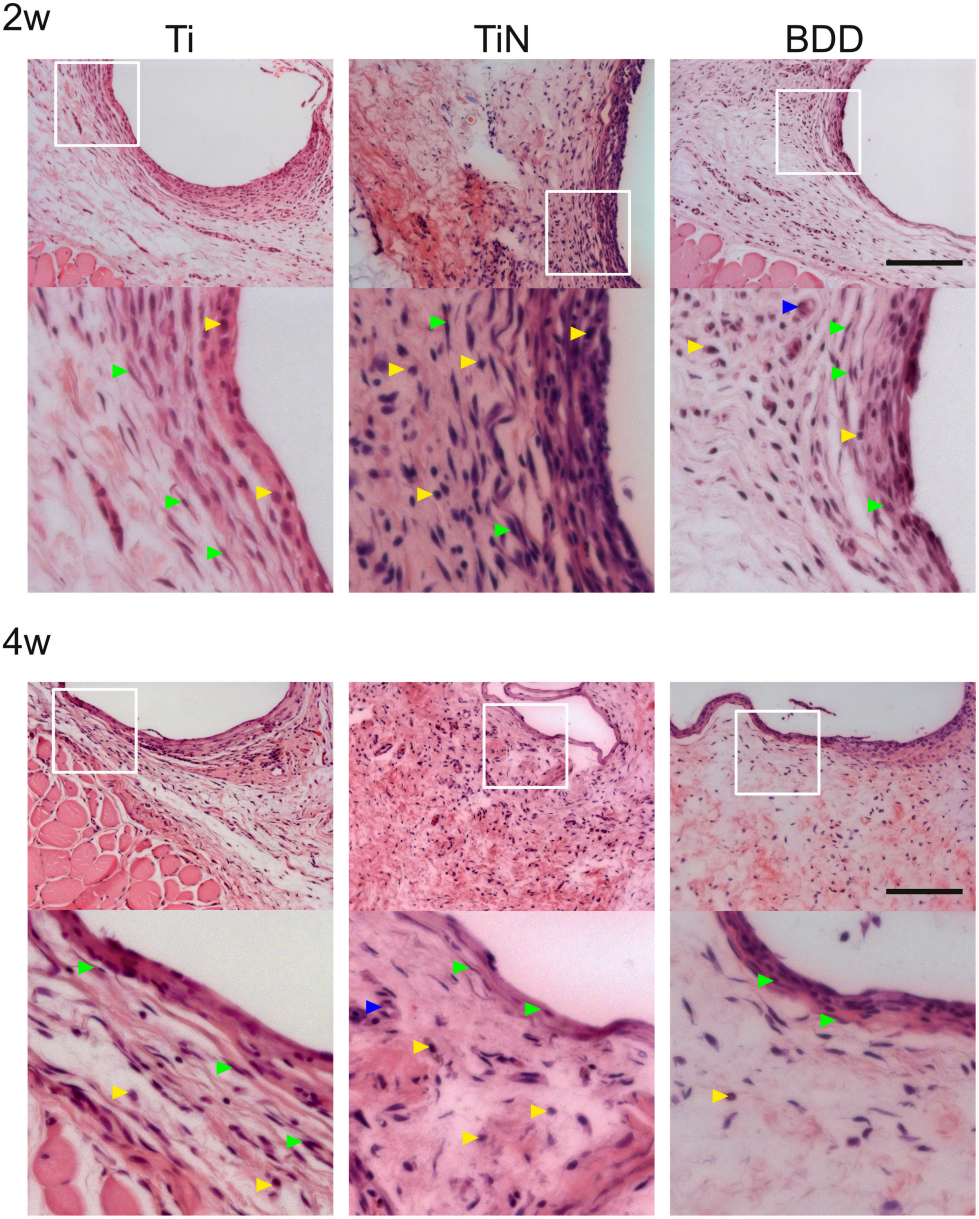
**Hematoxilin and eosin stained sections of the tissue adjacent to the subcutaneously implanted electrodes**. The overview images show the histological appearance of the pericapsular connective tissue after 2 and 4 weeks. For better visualization of the cells adjacent to the implants, magnified pictures of the area delimited by the white squares are presented below each overview image. Arrowheads indicate fibroblasts (green), inflammatory cells (yellow), and blood vessels (blue). Scale bar denotes 400 μm.

**Figure 3:**
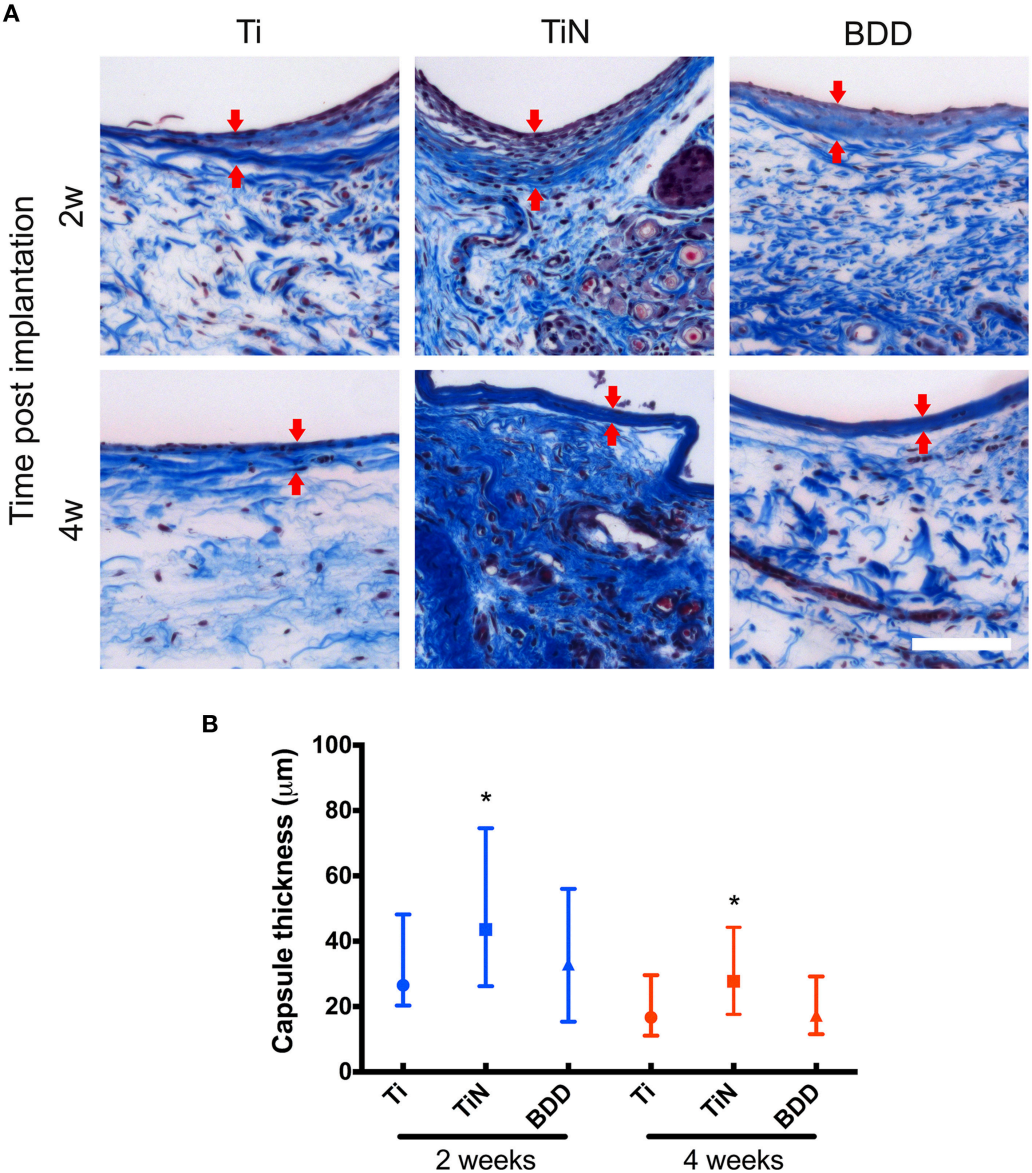
**Assessment of fibrous capsules formed around the implants. (A)** The Masson's trichrome staining highlights in dark blue the collagen fibers adjacent to the implants. Arrows indicate the boundaries of the fibrous capsules. Scale bar denotes 100 μm. **(B)** The thickness of fibrous capsules is presented as median with interquartile range (25th to 75th percentile). The groups entail at least *n* = 50 measurements. Statistically significant differences (*P* < 0.01) are indicated with respect to bare Ti implants (^*^).

The histological examination of the HE stained sections revealed a fairly uniform tissue reaction to the implanted materials (Figure 2). At 2 weeks, all implants evidenced the formation of thin capsules, consisting of a layer of collagen fibers aligned parallel to the implant surface, with variable presence of fibroblasts and some inflammatory cells. After 4 weeks, the number of cells surrounding the implants seemed to decrease, while the collagen fibers at the interface appeared to be denser. This observation was more evident for capsules around Ti and BDD implants. Image analysis of MT stained sections enabled assessment of collagen deposition and quantification of the fibrous capsule thickness (Figure 3). As shown in Figure 3B, the median thickness of the capsules consistently decreased with the implantation time. This decrease was statistically significant for all the implanted materials (*p* < 0.01). While the capsules formed around the BDD implants were equivalent to those on Ti, the capsules around TiN were significantly thicker at both time points (*p* < 0.01).

**Figure 4:**
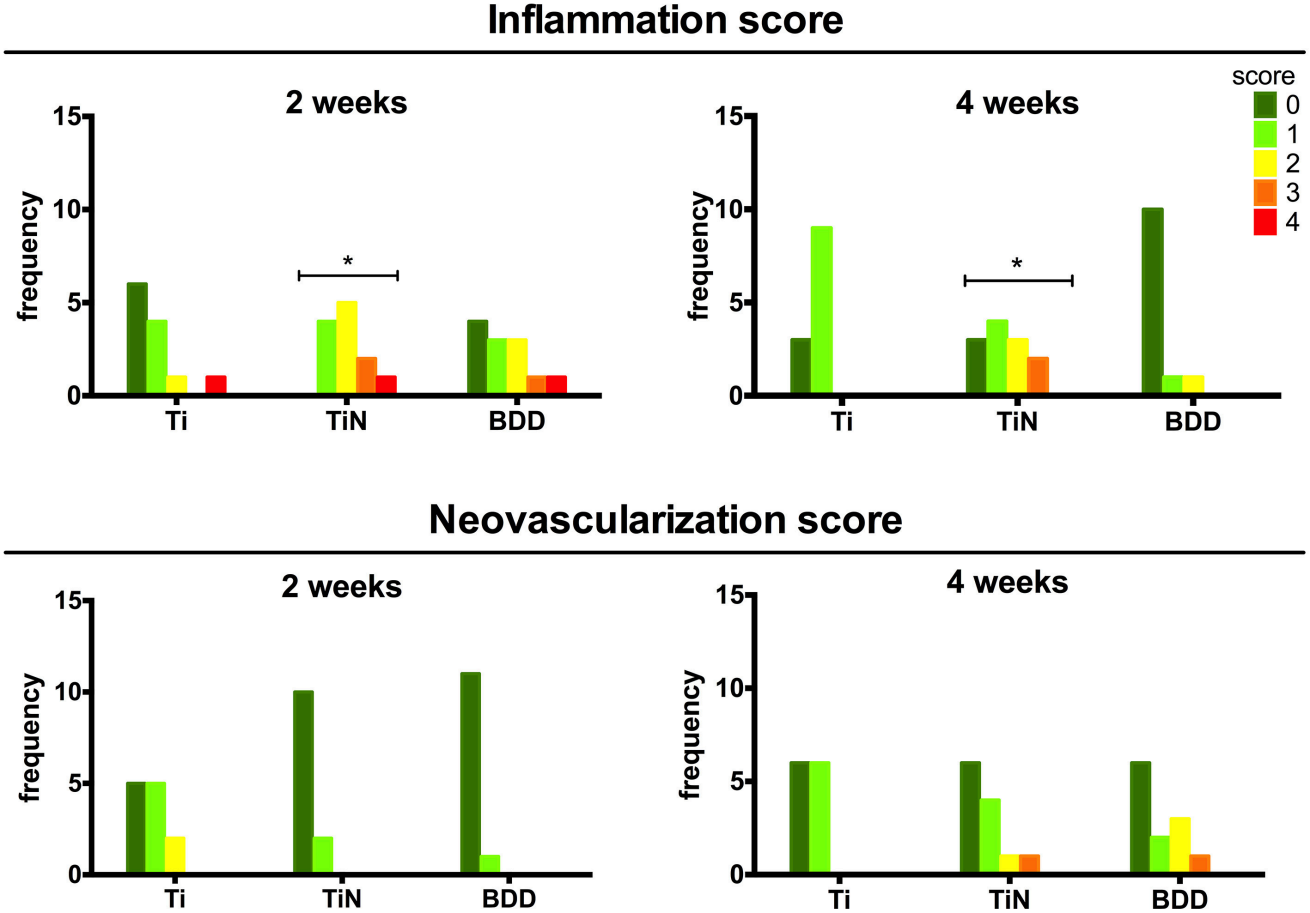
**Distribution of histomorphometric assessment scores**. The bar height indicates the number of images assigned to each score category. Statistically significant differences between distributions (*P* < 0.01) are indicated with respect to bare Ti implants (^*^).

Analysis of high magnification images at the implant interface allowed the scoring of inflammatory cells representing the different phases of the foreign body reaction as well as the presence of blood vessels. No giant cells or necrotic areas were observed for any of the implants. Figure 4 shows the frequency distributions for the inflammation scores obtained following the criteria presented in the Supplementary Table I. The scores obtained for the BDD samples represent a minimal tissue reaction, which was not significantly different from that obtained on the Ti implants. On the other hand, the TiN implants elicited a minimal to mild inflammatory reaction at both time points, which represent a significantly higher inflammatory activity than Ti (*p* < 0.01). Regarding the neovascularization scores, although both TiN and BDD seemed to display an increased vascularization density after 4 weeks, no statistically significant differences were found for any of the implants types.

## Discussion

The aim of this work was to assess the *in vivo* biocompatibility of BDD electrodes using a subcutaneous implantation model that is typically used to test for local effects after implantation. The electrodes were implanted through relatively small openings, to reduce the implantation time and the tissue damage caused by insertion. It is therefore assumed that the results of this study reflect reasonably well the foreign body response to BDD electrodes after implantation using minimally invasive techniques. TiN has been chosen as a reference material since it is widely used as coating for neural interfacing electrodes and also cardiac pacing leads (Schaldach et al., 1990; Weiland et al., 2002; Cogan, 2008).

The growth conditions for the BDD films were chosen as in a previous study, to obtain electrochemical parameters suitable for neural stimulation purposes (Meijs et al., 2013). In particular, films possessed an average impedance of 200 ohms at 1 kHz, which was consistent with their high boron content (Meijs et al., 2013). Boron incorporation was also evident form the characteristic B related peaks at 500 and 1230 cm^−1^ in the Raman spectrum (Prawer and Nemanich, 2004) and by the down shifting of the sp^3^ diamond related peak (Taylor et al., 2014). As revealed by the surface analysis, the CVD synthesis produced homogeneous and high quality BDD films on the electrodes, in agreement with previous studies using the microwave plasma enhanced linear antenna deposition system (Taylor et al., 2014; Taylor A. et al., 2015). The homogenous distribution of diamond crystallites also indicates the high cohesion of the synthesized films. The Ti6Al4V alloy has been previously reported to constitute an excellent substrate for the deposition of homogeneous and cohesive NCD films, thanks to the formation of a titanium carbide layer, which serves as precursor for the nucleation and growth of crystals during CVD (Booth et al., 2011). In particular, boron doped NCD films have been previously reported to exhibit high adhesion strength to Ti6Al4V substrates, displaying a resistance to delamination at normal stresses above 2 GPa (Liang et al., 2009). The high sp^3^/sp^2^ ratio also supports a strong coating adhesion, as a relatively high content of graphite in NCD films is usually associated with poor tribological properties and film delamination (Catledge et al., 2013).

The histological analysis of the HE and MT stained sections showed that BDD implants exhibited significantly thinner fibrous capsules and lower inflammation scores at both time points as compared to the TiN counterparts. An increased density of blood vessels around implants might suggest the persistence of granulation tissue at the interface; however, we did not observe any significant difference amongst the different implants. Our results are in agreement with a study from Garrett et al, who have reported reduced fibrous encapsulation to BDD implants after 4 weeks as compared to silicone polymer. The median thicknesses reported by their study are, however, slightly larger than the ones obtained here (86 vs. 18 μm). Since the magnitude and severity of the foreign body reaction is affected by several parameters, this difference might be explained by the different volume and shape of the implants. The effect of these parameters on the foreign body reaction has been described for several types of neural electrodes implanted in different locations (Szarowski et al., 2003; Polikov et al., 2005; Seymour and Kipke, 2007; Ortiz-Catalan et al., 2012). The apparent delayed healing response to the TiN implants observed here is consistent with a previous study by Satomi et al. who compared the tissue reaction to various Ti based materials using a rat subcutaneous implantation model (Satomi et al., 1988). They have shown that the subacute tissue response to TiN was less favorable than for pure Ti implants, as evidenced by the presence of granulation tissue around TiN implants after 3 and 7 days. After 14 days, the process of fibrous encapsulation was still not complete for TiN and only after 84 days the capsules were equivalent for all the implanted materials (Satomi et al., 1988).

Overall, our results reveal that the foreign body response to BDD appears to be essentially equivalent to that observed for the Ti alloy implants. Pure Ti and its alloys spontaneously build up a stable and inert oxide layer, which is associated with a slight inflammatory reaction upon implantation and a thin fibrous encapsulation over the course of a few weeks. The minimal tissue reaction to the BDD electrodes might be in part explained by its hydrogen terminated surface, providing an inert non-polar surface chemistry which is resistant to fouling by proteins (Shin et al., 2005). We have recently compared *in vitro* the protein adsorption and fibroblast adhesion to hydrogen terminated undoped and boron doped diamond films (Pennisi et al., 2014). Our study revealed that both intrinsic and boron-doped films grown on textured Ti substrates displayed resistance to protein adsorption and a slightly enhanced proliferation rate in comparison to bare Ti surfaces. Correspondingly, another factor affecting the foreign body reaction is the ability of the implant surface to promote the adhesion of cells involved in the wound healing process. In subcutaneous implants, an increased connective tissue attachment to the biomaterial surface is associated with decreased inflammatory response and fibrous encapsulation (Jensen et al., 2012). This correlation has also been shown for implants with intrinsic nanocrystalline diamond coatings, indicating that surface wettability and termination play an important role mediating the foreign body reaction (Kloss et al., 2011).

In this work we have for the first time synthesized high-quality BDD films on neural electrodes for an *in vivo* biocompatibility assessment in a rat subcutaneous implantation model. In summary, as compared to conventional TiN electrodes, BDD electrodes developed thinner fibrous capsules and elicited lower local inflammation scores, indicating that the foreign body reaction to the material was milder and had a faster resolution rate. The overall local tissue response to BDD implants was not significantly different than that of the control TiAlV alloy. The surface properties offered by diamond films may contribute to reduce the adsorption of serum proteins and promote cellular attachment *in vivo*, consequently minimizing the cascade of inflammatory events leading to foreign body reaction. The reduced protein fouling characteristics and the presence of a thinner fibrous encapsulation layer indicates that BDD electrodes may present a reduced impedance pathway for the transmission of electrical signals. In perspective, BDD films might provide electrodes providing safe and stable performance over time for chronic implantable neural prostheses and brain-computer interfaces.

## Author contributions

Conceived and designed the experiments: MA, AT, MF, CP. Performed the experiments: MA, AT, CP. Analyzed the data: MA, AT, CP. Contributed with reagents/materials/analysis tools: AT, MF, VZ, CP. Contributed to the drafting and reviewing of the manuscript: MA, AT, MF, VZ, CP.

### Conflict of interest statement

The authors declare that the research was conducted in the absence of any commercial or financial relationships that could be construed as a potential conflict of interest. The handling Editor declared a past collaboration in an EU funded project titled MERIDIAN (Micro and Nano Engineered Bi-Directional Carbon Interfaces for Advanced Peripheral Nervous System Prosthetics and Hybrid Bionics) with the author CP and states that the process nevertheless met the standards of a fair and objective review.

## Supplementary material

Supplementary Figure 1 **Approach for histological sectioning**. Macroscopic view of an implanted BDD electrode before tissue fixation seen from the ventral side. The histological sections were obtained perpendicularly across the electrode head, as indicated by the dotted line. The displayed histological section is oriented with the ventral side to the right.

Supplementary Table I **Criteria used to evaluate the severity of the inflammatory response**.

Supplementary Table II **Criteria used to evaluate the degree of neovascularization**.
