# Supplementary figures and images for: Boron-Doped Nanocrystalline Diamond Electrodes for Neural Interfaces: In vivo Biocompatibility Evaluation
Source: Front Neurosci. 2016 Mar 8;10:87. doi: 10.3389/fnins.2016.00087 (PMC4781860; doi:10.3389/fnins.2016.00087)

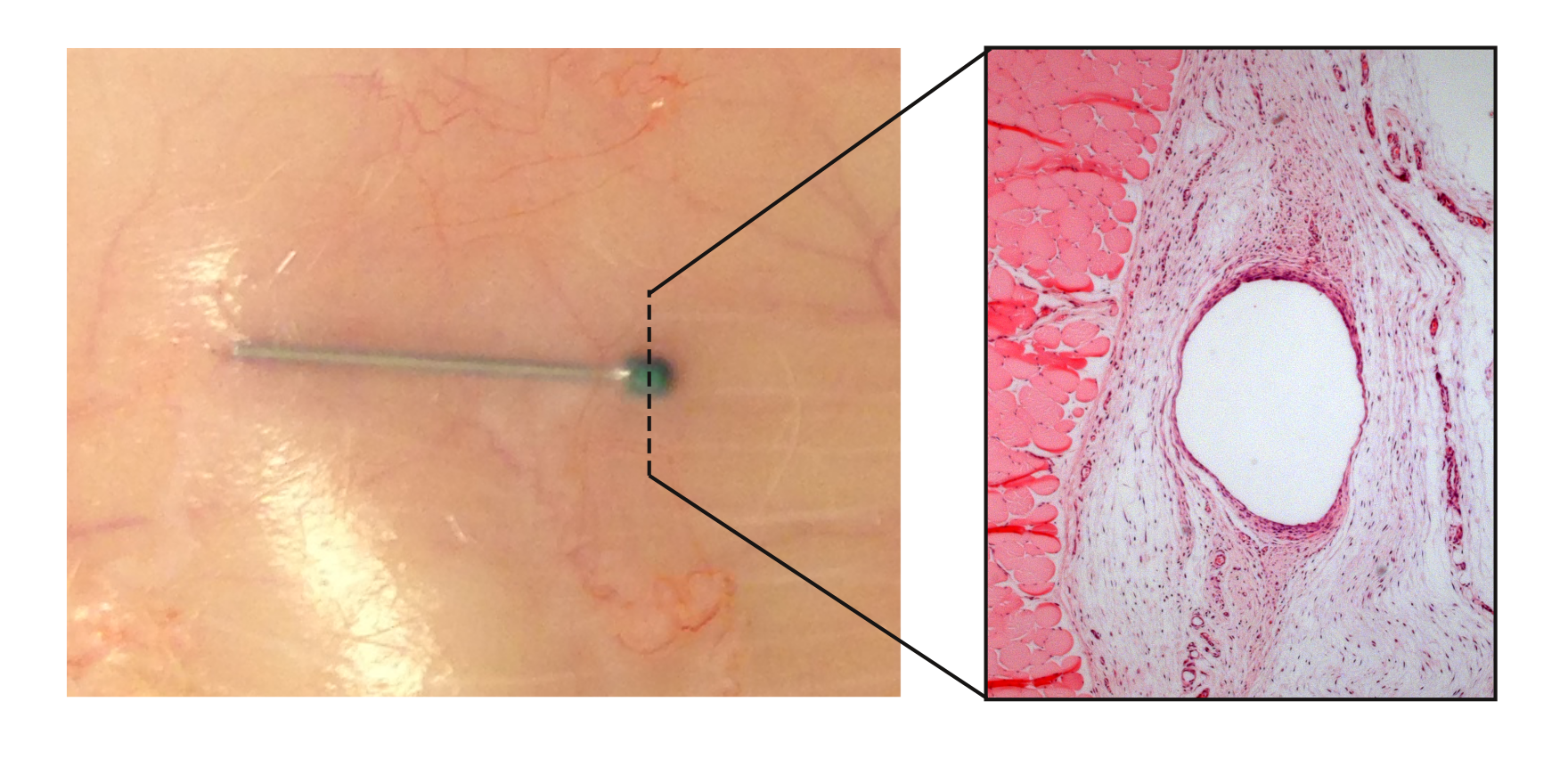

Supplement: Supplementary Figure 1 — Approach for histological sectioning. Macroscopic view of an implanted BDD electrode before tissue fixation seen from the ventral side. The histological sections were obtained perpendicularly across the electrode head, as indicated by the dotted line. The displayed histological section is oriented with the ventral side to the right. [file Image1.TIF]
