# Supplementary material for: Boron-Doped Nanocrystalline Diamond Electrodes for Neural Interfaces: In vivo Biocompatibility Evaluation
Source: Front Neurosci. 2016 Mar 8;10:87. doi: 10.3389/fnins.2016.00087 (PMC4781860; doi:10.3389/fnins.2016.00087)
Supplement: Supplementary Table I — Criteria used to evaluate the severity of the inflammatory response. [file Table1.DOCX]

**Supplementary Table I.** Criteria used to evaluate the severity of the inflammatory response.

|  | Score | | | | |
| --- | --- | --- | --- | --- | --- |
|  | 0 | 1 | 2 | 3 | 4 |
| Cell Type |  |  |  |  |  |
| Polymorphonuclear cells | None | Rare,  1-4/phf* | 5-10/phf | Heavy infiltrate | Packed |
| Lymphocytes | None | Rare,  1-4/phf | 5-10/phf | Heavy infiltrate | Packed |
| Plasma Cells | None | Rare,  1-4/phf | 5-10/phf | Heavy infiltrate | Packed |
| Macrophages | None | Rare,  1-4/phf | 5-10/phf | Heavy infiltrate | Packed |
| Giant Cells | None | Rare,  1-2/phf | 3-5/phf | Heavy infiltrate | Sheets |
| Necrosis | None | Minimal | Mild | Moderate | Severe |

*phf denotes per high-powered field.

|  |  |  |  |
| --- | --- | --- | --- |
