# Supplementary material for: Boron-Doped Nanocrystalline Diamond Electrodes for Neural Interfaces: In vivo Biocompatibility Evaluation
Source: Front Neurosci. 2016 Mar 8;10:87. doi: 10.3389/fnins.2016.00087 (PMC4781860; doi:10.3389/fnins.2016.00087)
Supplement: Supplementary Table II — Criteria used to evaluate the degree of neovascularization. [file Table2.DOCX]

**Supplementary Table II.** Criteria used to evaluate the degree of neovascularization.

|  | Score | | | | |
| --- | --- | --- | --- | --- | --- |
|  | 0 | 1 | 2 | 3 | 4 |
| Presence of vessels | None | Minimal, 1-3 buds | 4-7 capillaries | Broad capillary band | Extensive band |
